# Rifles and shotguns have similar animal welfare outcomes during aerial culling of non-native fallow deer (*Dama dama*)

**DOI:** 10.1017/awf.2025.10037

**Published:** 2025-09-18

**Authors:** David M Forsyth, Andrew J Bengsen, Andrew L Perry, Lee Parker, Mal Leeson, Jordan O Hampton

**Affiliations:** 1 Vertebrate Pest Research Unit, NSW Department of Primary Industries and Regional Development, Orange, NSW, Australia; 2 School of Biological, Earth & Environmental Sciences, University of New South Wales, Sydney, NSW, Australia; 3 Vertebrate Pest Research Unit, NSW Department of Primary Industries and Regional Development, Calala, NSW, Australia; 4 Ecotone Wildlife Veterinary Services, Inverloch, VIC, Australia; 5 Central Tablelands Local Land Services, Mudgee, NSW, Australia; 6 School of Veterinary Medicine, Murdoch University, Murdoch, WA, Australia; 7 Faculty of Science, University of Melbourne, Parkville, VIC, Australia

**Keywords:** Animal welfare, chase time, helicopter-based shooting, shooting, wildlife culling, wounding

## Abstract

Helicopter-based shooting using either a .308 semi-automatic rifle or a semi-automatic 12-gauge shotgun is widely used to manage non-native ungulate populations in Australasia, but the animal welfare outcomes of these two firearms have not been robustly compared. We conducted a randomised field study to compare the animal welfare outcomes of helicopter-based shooting of fallow deer (*Dama dama*) using a shotgun with three types of lead-based shot (Winchester^®^ 00 Buck, 1 Buck or 4 Buck) relative to a .308 rifle with 135-grain lead-based bullets in New South Wales, Australia, in 2023. All deer that were shot at (n = 390) were killed. Time-to-event curves for times from pursuit to first shot, first shot to insensibility, and the sum of these two metrics (‘total time’), were similar among the four ammunition types. The mean number of shots fired per deer was similar across all four ammunition types, but the mean number of wound tracts per deer increased across the four ammunition types with the number of projectiles per cartridge. All deer subjected to post mortem examination had 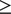 1 wound tract or projectile in the thorax. Our study indicates that using a .308 semi-automatic rifle or a 12-gauge semi-automatic shotgun for helicopter-based shooting of non-native deer, when the latter is used at ranges ≤ 30 m, provides similar animal welfare outcomes.

## Introduction

Helicopter-based shooting (hereafter ‘aerial culling’) has been used to control populations of non-native ungulates since the 1960s (Challies 1974). The method involves using a helicopter as a platform for finding and shooting ungulates. When an animal is sighted, the pilot manoeuvres the helicopter to within shooting distance and provides a stable shooting platform for the shooter, who sits either behind or adjacent to the pilot with the door removed (Challies 1974). Originally developed for recovering wild red deer (*Cervus elaphus*) carcases for commercial use (Challies 1974), the method has subsequently been used to cull many ungulate species (Hess & Jacobi 2011; Hampton *et al*. 2017; Cox *et al*. 2023).

A semi-automatic rifle chambered in .308 Winchester^®^ (almost identical to 7.62 × 51 mm NATO) has been widely used for aerial culling of ungulates in Australasia (Challies 1974; Senate Select Committee on Animal Welfare 1991). During the early 1980s, shooters targeting red deer for commercial recovery in New Zealand began to also carry a 12-gauge semi-automatic shotgun (‘shotgun’), because it was considered easier to place a killing shot in the head when deer were within 30 m, and a wound in the chest from a .308 rifle bullet would reduce the price paid for the carcase (M Beardsley, Department of Conservation, unpublished data). The .308 rifle was used for shooting deer at longer ranges. The shotgun is now commonly used for aerial culling of deer, Himalayan tahr (*Hemitragus jemlahicus*), feral goat (*Capra hircus*) and red-necked wallaby (*Notamacropus rufogriseus*) in New Zealand (M Beardsley, Department of Conservation, unpublished data). Australia does not have a history of aerial culling of ungulates for commercial carcase recovery, but shotguns have been used for aerial culling of feral pigs (*Sus scrofa*) since 1979 (T Korn, Independent Researcher, unpublished data) and have commonly been used to shoot feral goats (Feral Animal Aerial Shooting Team 2020). Shotguns have been used to shoot feral goats from helicopters in Hawai’i, USA (Burt & Jokiel 2011). In Southern Africa, shotguns are used from helicopters for commercial harvesting of impala (*Aepyceros melampus*) (Nkosi *et al*. 2022), and in North America, for culling of feral pigs (Lavelle *et al*. 2025).

Whereas a rifle fires one projectile (‘bullet’), a shotgun can fire many projectiles (‘pellets’). The shotgun ammunition most commonly used for aerial culling of ungulates in New Zealand contains nine lead pellets (Winchester^®^ 00 Buck) (M Beardsley, Department of Conservation, unpublished data), and in Australia contains between nine and 27 lead pellets (Feral Animal Aerial Shooting Team 2020; Bradshaw *et al*. 2023). These shotgun ammunition types retail as ‘buckshot’ and have a recommended effective maximum range of 30 m (Feral Animal Aerial Shooting Team 2020), much less than that of the .308 rifle (> 200 m; Hampton *et al*. 2022). The animal welfare outcomes of using a shotgun or rifle for culling have not, however, been evaluated in a way that controls for variables that include shooter, deer density, and landscape features such as terrain and the amount of vegetation cover that could potentially affect animal welfare outcomes (Hampton *et al*. 2021a).

Previous assessments of the animal welfare outcomes of aerial culling of ungulates have used similar field methods to quantify five metrics related to animal welfare outcomes. The five metrics are:

1. Chase time (CT): the time taken from the start of pursuit to firing the first shot at the animal (Jacques *et al*. 2009).
2. Time to insensibility (TTI): the time from the first shot being fired to that animal becoming insensible (i.e. the moment the animal becomes recumbent and ceases moving (Hampton *et al*. 2014, 2017; Smith & Ryeng 2022). This has also been referred to as ‘time to apparent death’ (Hampton *et al*. 2015; Bradshaw *et al*. 2023) and ‘time to incapacitation’ (McTee *et al*. 2017) but is most accurately described as ‘time to insensibility’ (Hampton & Forsyth 2016).
3. Total time (TT): the time from the start of pursuit to insensibility (i.e. TT = CT + TTI). This is the total duration of stress experienced by animals (Ponjoan *et al*. 2008) up to the time when they become insensible (Hampton *et al*. 2017).
4. Non-fatal wounding rate (NFWR): the proportion or percentage of animals that are shot and hit but subsequently escape. Non-fatal wounding is considered the worst animal welfare outcome for any culling operation because it causes protracted (but unmeasured) suffering (Aebischer *et al*. 2014).
5. Accuracy of the shooting and nature of the ballistic injuries (Hampton *et al*. 2021b). To assess this, the number and anatomical location of gunshot wound tracts are recorded (Hampton *et al*. 2017).

A limitation of the traditional approach to animal welfare assessment in shooting studies, and especially aerial culling studies, is that the animals are observed remotely. Hence, insensibility and death are presumed rather than confirmed. Ground-based inspections not conducted immediately after an animal is rendered insensible would also not detect animals that were hit and rendered temporarily insensible but then regained sensibility and mobility (Grandin 2002). Consequently, previous estimates of the frequency of non-fatal wounding relying upon delayed ground-based inspections of carcases could be biased low (Hampton *et al.* 2017).

Aerial culling of ungulates in Australia is guided by procedural documents (i.e. standard operating procedures, codes of practice and manuals; Hampton *et al*. 2016) that vary between states and territories. In the state of New South Wales (NSW), aerial culling conducted by state government agencies (i.e. Local Land Services and the NSW National Parks and Wildlife Service) is conducted according to the *Feral Animal Aerial Shooting Team (FAAST) Manual* (Feral Animal Aerial Shooting Team 2020). This document governs several variables of potential relevance to animal welfare outcomes, including firearm type (i.e. rifle or shotgun), calibre and bullet mass, and pilot and shooter training. A 12-gauge shotgun with SG (‘small game’) or SSG (‘special small game’) shot can be used for feral goat and feral pig at distances of ≤ 30 m (Feral Animal Aerial Shooting Team 2020). The firearm and ammunition currently approved by the Feral Animal Aerial Shooting Team to conduct aerial culling of fallow deer (*Dama dama*) is the FN SCAR®-H semi-automatic .308 rifle with 130- or 135-grain hollow-point lead bullets (Feral Animal Aerial Shooting Team 2020). Shotgun ammunition is not approved for use on any deer species during FAAST aerial culling programmes but is approved for use on feral goats (which are of similar size and mass to adult female fallow deer) and feral pigs. Each animal shot must be shot at least twice, with at least one bullet placed in the heart/lung of the animal, and before shooting further animals. If the shotgun is used, at least one of the shots must be centred on the heart/lung region (Feral Animal Aerial Shooting Team 2020).

The aim of this study was therefore to compare the animal welfare outcomes of aerial culling of fallow deer in NSW using a semi-automatic shotgun and three types of ‘buckshot’ ammunition with those outcomes when using a semi-automatic .308 rifle while controlling (as far as is practically possible) for individual shooter, deer density and concealing cover. Specifically, we quantified the five metrics described above. To maximise the animal welfare outcomes of aerial culling, NFWR and TT should be minimised.

## Materials and methods

### Ethical approval

This research was performed in accordance with the Australian code for the care and use of animals for scientific purposes 8th edition 2013 (updated 2021), with approval granted by the NSW Department of Primary Industries Orange Animal Ethics Committee (permit OAEC-0504) and the NSW Feral Animal Aerial Shooting Team Committee.

### Study area and species

We conducted our study on agricultural properties in the eastern Liverpool Plains and western Liverpool Range, Northern Slopes region, NSW, Australia (Davis *et al*. 2023). Briefly, the area consists of steep to undulating grazing country and black-soil plains used for cropping (cotton, maize, sorghum, canola, wheat and barley) surrounded in the east by the western side of the Great Dividing Range and in the south by the Liverpool Ranges. The vegetation on the steeper and higher-elevation lands is dominated by modified native grasslands used for livestock (cattle and sheep) grazing, rising up into native Grassy Woodlands, with patches of Northern Warm Temperate Rainforest near the Great Dividing Range, and occasional Sclerophyll Woodlands in the drier west (New South Wales Office of Environment and Heritage 2017). Fallow deer are the most common non-native deer species in this area. Aerial culling of deer was conducted in our study area in 2018 and 2019 (site ‘NSW4’ in Bengsen *et al*. 2022), with the objective of reducing competition for food with domestic livestock during severe drought (Davis *et al.* 2023). The culling reduced the population to approximately 23.4 deer per km^2^ in 2019 (Bengsen *et al*. 2022). The animals in this study were part of a planned aerial cull and would have been shot irrespective of whether or not they were subjects in the current study. An aerial survey during 13–17 February 2023 (i.e. immediately preceding our trial) estimated that the population density of fallow deer was 37.5 deer per km^2^ (95% CI: 25.3–55.8; Supplementary material S1).

Fallow deer are strongly sexually size dimorphic, with older males attaining twice the mass of adult females (Chapman & Chapman 1997). In Australia, fallow deer give birth during November–December (Bentley 1995), and the single offspring becomes independent at 3–4 months (Mulley 2007). Males > 1 year old commonly live apart from females (i.e. are ‘spatially segregated’) outside of the breeding season (Davis *et al.* 2023) which occurs during March and April in Australia (Bentley 1995). In our study area, use of tree cover by fallow deer fitted with GPS-tracking collars during our trial varied by sex (Bengsen *et al*. 2024). During daylight hours, adult females used tree cover (*circa* 55–70% use) more than did adult males (*circa* 30–50% use).

### Treatments and control

Our objective was to determine how TT and NFWR differed when using the shotgun relative to the .308 rifle. Our control was therefore lead-based 135-grain hollow-point ammunition (Outdoor Sporting Agencies, Tarneit, VIC, Australia) fired from a FN SCAR^®^-H semi-automatic rifle chambered in .308 Winchester^®^ (Fabrique National Herstal, Herstal, Belgium) and fitted with a non-magnified red-dot scope (i.e. current practice in FAAST-mandated fallow deer aerial culling programmes in NSW).

Our treatments were three types of SG shotgun ammunition fired from a Benelli M4 semi-automatic 12-gauge shotgun (Benelli Armi SpA, Urbino, Italy) with a 50-cm barrel, full choke, tubular magazine with a capacity of seven rounds (providing a total capacity of eight rounds), non-telescopic pistol grip stock, and an Aimpoint^®^ Micro red-dot scope with zero-magnification (Manassas, Virginia, USA). The ammunition types were all lead-based: (1) Winchester^®^ SuperX™ 70-mm 00 Buck (nine lead pellets); (2) Winchester^®^ SuperX™ 70-mm 1 Buck (16 lead pellets); and (3) Winchester^®^ SuperX™ 70-mm 4 Buck (27 lead pellets). Shotgun pellets made from lead, being a soft metal, often deform and fragment upon impact within the bodies of shot animals (Green *et al.* 2022). In recognition of the threat posed by toxic lead ammunition (Katzner *et al.* 2024), non-toxic metals are also used to manufacture shotgun pellets, including steel (Pierce *et al.* 2015), tungsten and bismuth (Kraabel *et al.* 1996).

The firearm is that prescribed for use in NSW state agency aerial culling (Feral Animal Aerial Shooting Team 2020). Winchester^®^ 00 Buck ammunition is widely used for aerial culling of ungulates in New Zealand (M Beardsley, Department of Conservation, unpublished data), and the Winchester^®^ 1 Buck ammunition has been used for aerial culling of fallow deer in South Australia (Bradshaw *et al*. 2023). The 00 Buck ammunition has the greatest kinetic energy per pellet but the fewest pellets, the 4 Buck ammunition has the least kinetic energy per pellet but the most pellets, and the 3 Buck ammunition is intermediate in kinetic energy per pellet and number of pellets. Fallow deer shot with 00 Buck ammunition would be expected to have the fewest pellets enter the head or thorax, but those pellets would be expected to penetrate the deepest. In contrast, fallow deer shot with 4 Buck ammunition would be expected to have the most pellets enter the head or thorax, but those pellets would be expected to penetrate the least. Fallow deer shot with 1 Buck ammunition would be expected to have numbers of pellets entering the head or thorax and penetration depths intermediate between the 00 Buck and 4 Buck ammunition. It is unclear how this trade-off will affect chase times as, to our knowledge, no study has reported this for any deer or other ungulate species.

Our study population was wild, free-living fallow deer occupying a variety of habitats, and hence we could not *a priori* assign individual deer to a treatment or control group: rather, individual deer were included in the one ammunition type used in each sortie (2-h intervals during which culling was conducted). Hence, our study design was different to the randomised controlled trials commonly used in clinical medicine (Kendall 2003) and different to that used to assess the animal welfare outcomes of two bullet designs for killing young harp seals (*Pagophilus groenlandicus*) in Norway (Ryeng & Larsen 2021). If only one shotgun ammunition type were included in the trial, then we could have randomly assigned encountered deer to treatment or control, with the shooter switching between .308 rifle and shotgun within each sortie. However, because we had three shotgun ammunition treatments, we considered that the practical difficulties of implementing that approach in our trial were too great. Therefore, we randomly assigned one ammunition type to a sortie without replacement until all four ammunition types were included, repeating until the desired sample size was attained. We believe that this design best met the objective of minimising systematic differences between the treatment and control groups in factors, known and unknown, that could affect outcomes.

### Sample size calculations

The desired sample size for each of the three treatments and the control were guided by a sample size calculation (Supplementary material S2). Briefly, we wanted to detect increases rather than decreases in TT relative to the control, because that would indicate an increase in the duration of stress prior to animals being killed. There are no established guidelines or standards for what constitutes an acceptable TT for aerial culling, but we considered that an increase of > 50% of the expected duration compared with current practice would be a strong indication that the shotgun causes unnecessary increased duration of stress for fallow deer subject to aerial culling. Previously estimated TTs using the .308 rifle with 135-grain bullets in NSW (i.e. the same as the control in this trial) were log-normally distributed with a median of 133 s, a geometric mean of 148 s and 5th and 95th percentiles of 50 and 700 s, respectively (Hampton *et al*. 2021a). Our simulations indicated that a sample size of 100 animals per group would provide a 78% probability of detecting an increase of 48 s or 33% relative to those previous data for the .308 rifle with 135-grain ammunition. The same sample size would provide 91 and 98% power for effect sizes of 60 and 74 s, respectively (Table A in Supplementary material S2), under the assumed treatment effects. We therefore sought a sample size of 100 fallow deer in each of the three treatments and the control group, to make robust conclusions about TTs. For further details, see Supplementary material S2.

### Aerial culling procedure

The aerial culling occurred during 20–23 February 2023 and was conducted according to FAAST procedures (Feral Animal Aerial Shooting Team 2020). The aircraft was an Airbus AS350 B2 Écureuil (‘Squirrel’) helicopter (Airbus, Marignane, France), with the pilot accredited by FAAST to conduct aerial culling. A navigator sat alongside the pilot to (i) search for flying hazards, (ii) ensure that the aircraft targeted deer that were inside the designated shoot area, and (iii) record kills on a tablet. The shooter had been a FAAST-accredited shooter for 23 years and prior to this trial had 45,320 kills, including 4,150 fallow deer with the .308 semi-automatic rifle and approximately 12,000 feral pigs and 4,000 feral goats with the shotgun. The shooter sat in the rear, directly behind the pilot. An independent veterinarian sat in the rear alongside the shooter.

Between sorties the helicopter was refuelled and additional ammunition loaded into the aircraft. A maximum of 6 h of culling was conducted on any day. The number of sorties needed to achieve the desired sample size for each deer would strongly depend on the density of deer encountered (Bengsen *et al*. 2022), and we anticipated that two to four sorties would be needed per treatment. The order in which the treatments and the control were applied was determined randomly within each sequence of four sorties (Supplementary material S3), and only one treatment was applied in each sortie to eliminate the possibility of mixing the treatment (i.e. shotgun ammunition) types. On detecting a fallow deer in the designated shoot area, the shooter and pilot communicated until the aircraft was positioned such that a safe and effective shot could be taken by the shooter.

Following the FAAST manual (Feral Animal Aerial Shooting Team 2020), the shooter targeted the heart-lung region (i.e. thorax). There was repeat shooting of deer, with at least one shot to the thorax or, if not possible due to the position of the animal, the head (Feral Animal Aerial Shooting Team 2020). A fly-back procedure was also prescribed to confirm that an animal that had been shot was dead. If there was uncertainty as to whether the deer was dead, then a further shot was directed into the thorax or head. Following the FAAST manual (Feral Animal Aerial Shooting Team 2020), to minimise the risk of dependent fawns being orphaned, this age class was targeted first. If female deer were shot, the helicopter searched nearby for any dependent fawns which were then targeted by the shooter.

### Helicopter-based observations

When a fallow deer (hereafter ‘deer’) was detected, the veterinarian used a digital voice recorder to record the time that the pursuit started (to the nearest second), the time that a shot was fired, the outcome of the shot (miss, hit to head, hit to thorax, hit to other area [specify which other area]). It is possible that some hits may not have been detected and recorded as misses. After mandatory repeat shooting was completed (time recorded and noted as ‘repeat shoot’) and where the pilot considered it safe, the helicopter was landed as close as possible to the recumbent deer to allow ground inspection by the veterinarian (see *Ground-based observations* below). We quantified CT, TTI and TT (all in seconds) from the voice recordings.

### Ground-based observations

**Figure 1.**
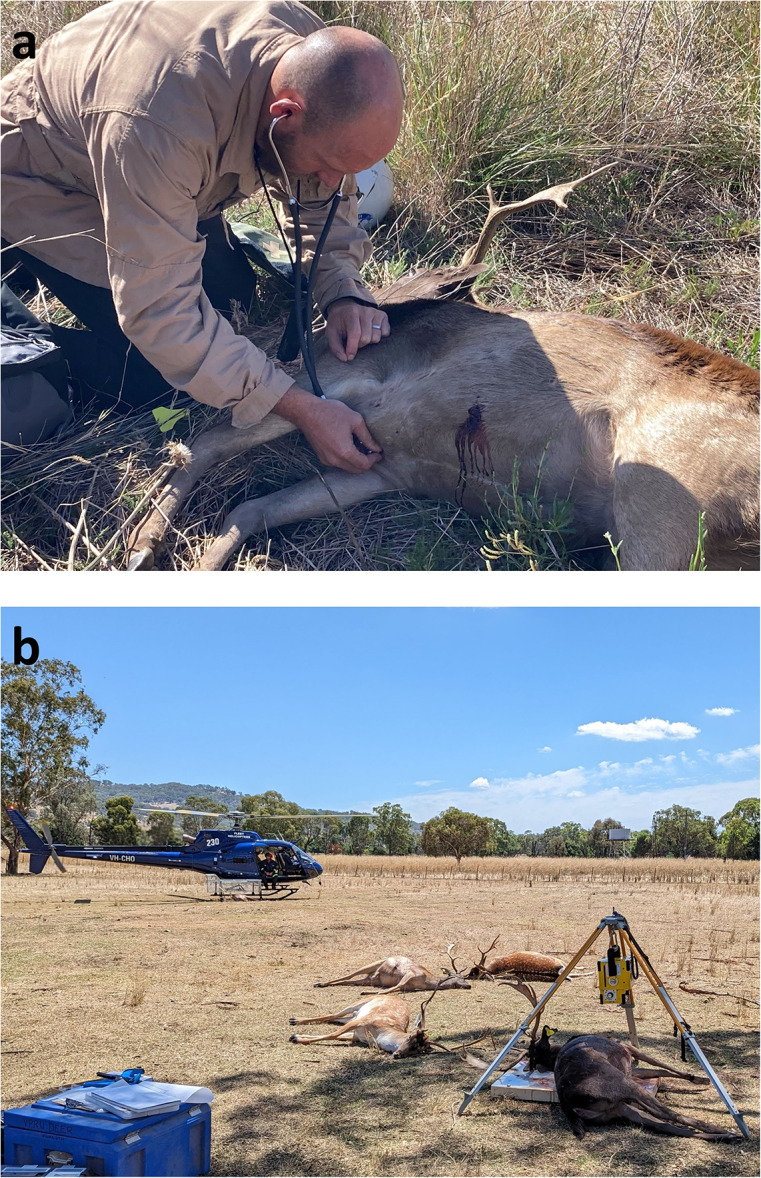
Field methods used to collect post mortem data during our study of aerial culling of fallow deer (*Dama dama*) using four ammunition types in New South Wales, Australia, showing (a) *in situ* assessment of sensibility and death in an adult male by an independent veterinarian; note the wound tract in the cranial ventral abdomen and the yellow ear-tag that will be attached prior to transport for *ex situ* inspection (image: M Leeson) and (b) four adult males ready for *ex situ* assessment, with one on the portable digital radiography system; note the helicopter (with skid basket attached) used for aerial culling (image: A Bengsen).

The veterinarian, after recording the time, assessed *in situ* whether the recumbent deer was insensible (corneal reflexes) and dead (i.e. no heartbeat detected with a stethoscope) (DeNicola *et al*. 2019) (Figure 1[a]). The dead deer was then sexed (by external genitalia and, for yearling and adult males, by the presence of antlers) and aged (adult, yearling or fawn) by body size and the pattern of tooth eruption (Fraser & Sweetapple 1993). A uniquely coded cattle tag was attached to one of the deer’s ears. If the pilot deemed it safe to do so, then the dead deer was placed in a ‘skid basket’ (a metal cage fitted to the skid of the helicopter) and transported to a processing area for *ex situ* post mortem assessment, either singly (for adult males) or with up to three other deer (for other sex-age classes) (Figure 1[b]).

At our processing area, deer were weighed to the nearest 0.5 kg with a Salter 100-kg clock-face hanging scale (Salter, Thomastown, VIC, Australia). We next used radiography to assess the terminal ballistic performance of the lead shotgun pellets and .308 lead bullets in deer tissues. Radiography is an accepted method for assessing terminal ballistic patterns in wildlife shooting (Broadway *et al.* 2020; Hampton *et al*. 2021b; Nkosi *et al*. 2022). An Exprimer EVS 2430 portable digital radiography system (DRTECH, Gyeonggi-do, Republic of Korea) was set at 80 kVp and 2.5 mAs and a wireless 25 × 30 cm digital plate used, with an Atomscope TR9020B (DLC Vet, Hoppers Crossing, Australia) portable veterinary X-ray generator suspended on a surveyor’s tripod (Hampton *et al*. 2023). Standardised lateral radiographs were taken of the left side of each deer, with the image centred on the middle of the thorax (for deer shot in the thorax), neck (for deer shot in the neck), or head (for deer shot in the head). Images were saved in DICOM format and interpreted by the veterinarian using the programme ImageJ (Green *et al*. 2022; Leontowich *et al*. 2022; Hampton *et al*. 2023). The presence/absence of whole pellets, and their anatomical location (head, neck, thorax, abdomen and limbs) was recorded. Finally, the lower jaw was removed for subsequent ageing by tooth eruption and cementum annuli (Fraser & Sweetapple 1993).

### Non-fatal wounding

Non-fatal wounding could have been detected from either helicopter-based observations (wounded and mobile animals) or ground-based observations (wounded but recumbent animals). Helicopter-based observations do not necessarily detect deer that are hit and rendered insensible but return to consciousness (Hampton *et al*. 2017). Our protocol minimised this possibility because, when a deer was shot at and hit, the helicopter followed it exclusively until the pilot pronounced the deer to be dead, whereupon the helicopter immediately landed as close to the deer (if safe to do so) and the veterinarian checked for a heartbeat. The NFWR was therefore the proportion of deer shot that were detected as either wounded and mobile or wounded and recumbent.

### Statistical analysis

For each deer at which 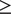 1 shot was fired, we classified the outcome of the encounter into one of four categories: (1) deer hit and rendered insensible 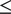 1 s after being shot; (2) deer hit and rendered insensible > 1 s after being shot; (3) deer hit and escaped wounded; and (4) deer not hit and escaped unwounded. We assumed that hits were accurately recorded, but it is possible that some may not have been detected and recorded as misses. We estimated the probability of each of these outcomes for each ammunition type using logistic regression, specifying ammunition type as a categorical covariate (Hampton *et al*. 2021a). We used survival functions to estimate CT, TTI and TT from the start of a chase to the point at which an animal was rendered insensible (TT). Some TTI observations could not be assigned a precise value, because the animal was not visible to the observer at the time that it was rendered insensible. In these cases, the minimum TTI was recorded (i.e. the data were right-censored). We fitted an exponential survival model (Hampton *et al*. 2021a) to the CT and TTI data, including ammunition type (*K* = 4) as a covariate in the likelihood function. TTI values for censored data were imputed by sampling an interval distribution spanning the minimum TTI observed for that datum and the maximum TTI across all data (Plummer 2017). TT was derived within the model as the sum of CT and the observed or imputed TTI for each observation. Survival functions were then fitted to TT using a second exponential model, again including ammunition type as a covariate. After examining the results, we repeated the process without the ammunition type covariate to estimate CT, TTI and TT across all four ammunition types. We used the deviance information criterion (DIC) to compare the balance of fit and complexity for the two models.

The mean number of wound tracts per deer was estimated for each ammunition type from the post mortem data using Poisson regression. The number of wound tracts was the response variable and ammunition type was the explanatory variable. We used the same process to estimate the proportions of shotgun pellets in each major anatomical zone, using the number of pellets counted in radiographic images as the dependent variable. We also used logistic regression to estimate the proportion of pellets that appeared to have missed deer for each shotgun ammunition type, using the difference between the expected number of pellets for each deer (i.e. number of shots fired × number of pellets per shot) and the number of pellets detected in radiography as the response variable.

All models were implemented in JAGS version 4.3.0 (Plummer 2017) called via the runjags package version 2.04-2 (Denwood 2016) in R version 4.0.3 (R Core Team 2022). We used normal priors 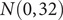 for parameters in the encounter and wound tract location models. For the survival models, we estimated exponential functions using Weibull distributions with the shape parameter fixed at 1 and a gamma prior 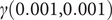 for the scale parameter. For all models, we used 10,000 MCMC draws from each of four chains after discarding 5,000 burn-in draws. Convergence and burn-in adequacy were assessed by examining trace plots, overlap of posterior distributions from each chain and the Gelman–Rubin statistic 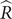 (Brooks & Gelman 1998). For all models, 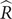 was ≤ 1.001. Parameter estimates are reported as posterior means and 90% highest posterior density intervals (HPDIs).

## Results

### Ante and post mortem sample sizes and demographics

We recorded ante mortem observations for a total of 390 fallow deer shot in 117 groups across 17 sorties (Table 1; Table A in Supplementary material S3). Sample sizes ranged from 94 to 100 for the four ammunition types but, due to the location of the deer relative to the observer, TTI could not be directly observed for 17–27% of animals in each of the four ammunition types (Table 1). Adult males comprised 51–88% of the ante mortem samples in each of the four ammunition types, and 68% of the total sample.

**Table 1.** Number of aerial culling sorties, ante mortem and post mortem (*in situ* and *ex situ*) observations per ammunition type, including the number of ante mortem observations in which time-to-insensibility (TTI) was not directly observed (i.e. censored)

Firearm/ammunition	Sorties	Ante mortem	TTI censored	Post mortem	
				In situ	Ex situ
.308 rifle	5	99	25	19	16
00 Buck	4	94	21	16	9
1 Buck	4	97	26	22	19
4 Buck	4	100	17	19	15
Total	17	390	89	76	59

Post mortem data were collected *in situ* for 76 deer (Figure 1[a]), and 59 of these were transported by the helicopter for *ex situ* assessment (Figure 1[b]) (Table 1). Adult males were the most common age-sex class for all ammunition types in the post mortem sample (58–100%), except for the 00 Buck ammunition, in which adult females and adult males comprised 67 and 22% of the sample, respectively.

### Body masses of fallow deer

The body masses of the 57 fallow deer that were weighed and aged by tooth eruption and cementum annuli ranged from 17 to over 100 kg ( 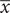 = 68.5 kg, SD = 20.4 kg; median = 65.0 kg, interquartile range [IQR] = 33.0 kg) (Table 2). The body masses of seven adult males exceeded what our 100-kg scales could measure, so the maximum and mean values for this age-sex class are biased low.

**Table 2.** Body masses of 57 fallow deer (*Dama dama*) shot during our trial

Age-sex class	*n*		Body mass (kg)	
		Mean (± SD)	Median	Minimum–maximum
Fawn female	1	19.0 (N/A)	19.0	19.0–19.0
Fawn male	1	17.0 (N/A)	17.0	17.0–17.0
Yearling female	2	42.5 (± 3.5)	42.5	40.0–45.0
Yearling male	25	61.1 (± 5.7)	61.0	50.0–70.0
Adult female	8	51.4 (± 3.4)	50.5	46.5–57.0
Adult male	20	92.2 (± 7.1)	92.0	81.0– >100.0

Animals were weighed entire. SD: standard deviation; N/A: not available as n = 1.

### Probability of shooting outcomes

**Figure 2.**
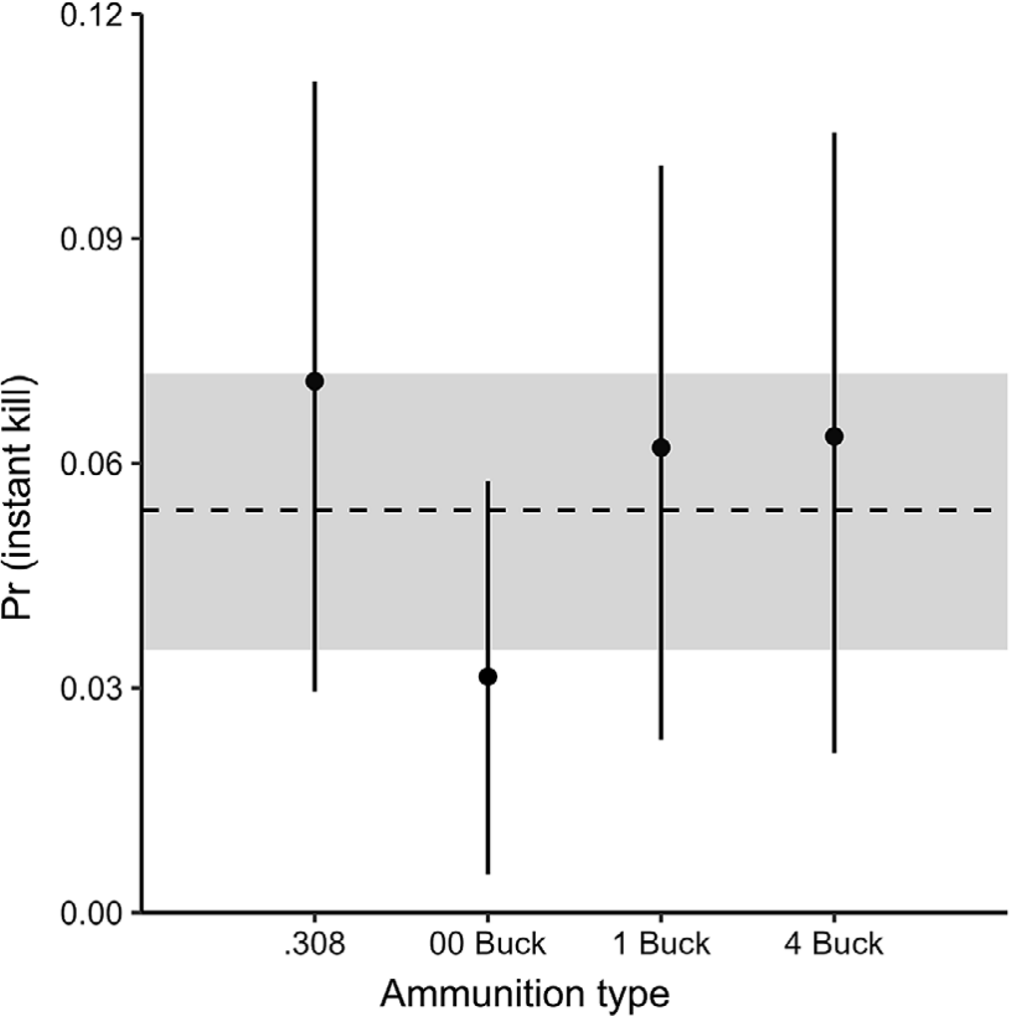
Probability of a fallow deer (*Dama dama*) being rendered insensible within 1 s (‘instant kill’) of being shot from a helicopter with one of three shotgun ammunition types or a .308 centrefire rifle. Vertical bars indicate 90% Highest Posterior Density Intervals (HPDIs). Dashed line and shaded polygon indicate the mean and 90% HPDI across all four ammunition types, respectively.

All 390 deer that were shot at were killed; none escaped, wounded or unwounded. Across all ammunition types, the probability of a deer being rendered insensible within 1 s of being shot (i.e. immediate insensibility) was 0.05. The greatest probability of immediate insensibility was for the .308 rifle (0.07), whereas deer shot with 00 Buck shotgun ammunition had the lowest probability of immediate insensibility (0.03; Figure 2). However, the probability that deer shot with the .308 rifle had a greater probability of immediate sensibility than deer shot with the 00 Buck ammunition was only 64%.

### Time-to-event parameters

**Figure 3.**
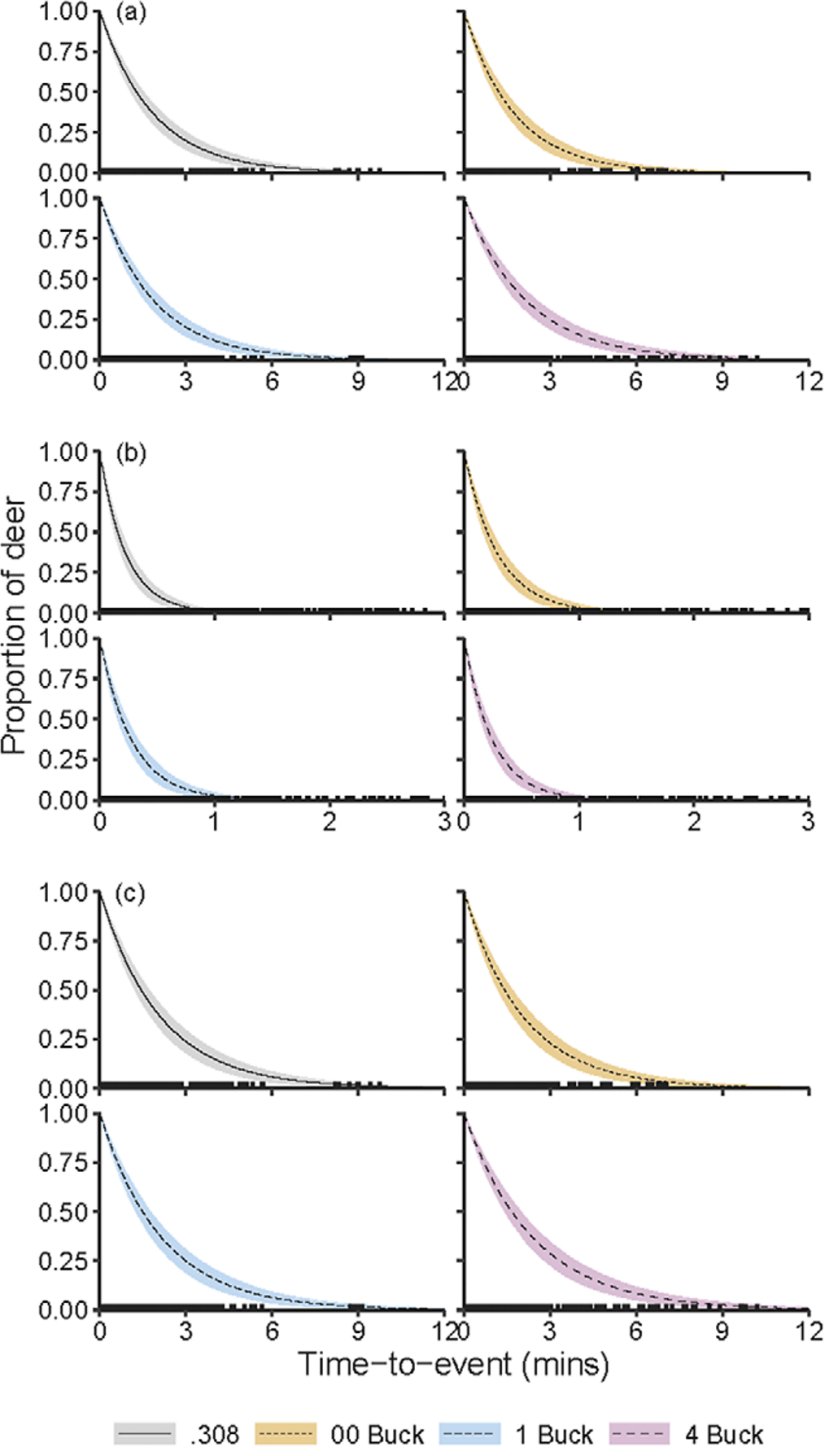
Exponential survival curves describing (a) chase time, (b) time between being shot at and being rendered insensible, and (c) total time from initial encounter to insensibility for fallow deer (*Dama dama*) subjected to aerial culling with one of four ammunition types in New South Wales, Australia. Solid lines and shading indicate the posterior means and 90% HPDIs, respectively. For sample sizes, see Table 1.

**Figure 4.**
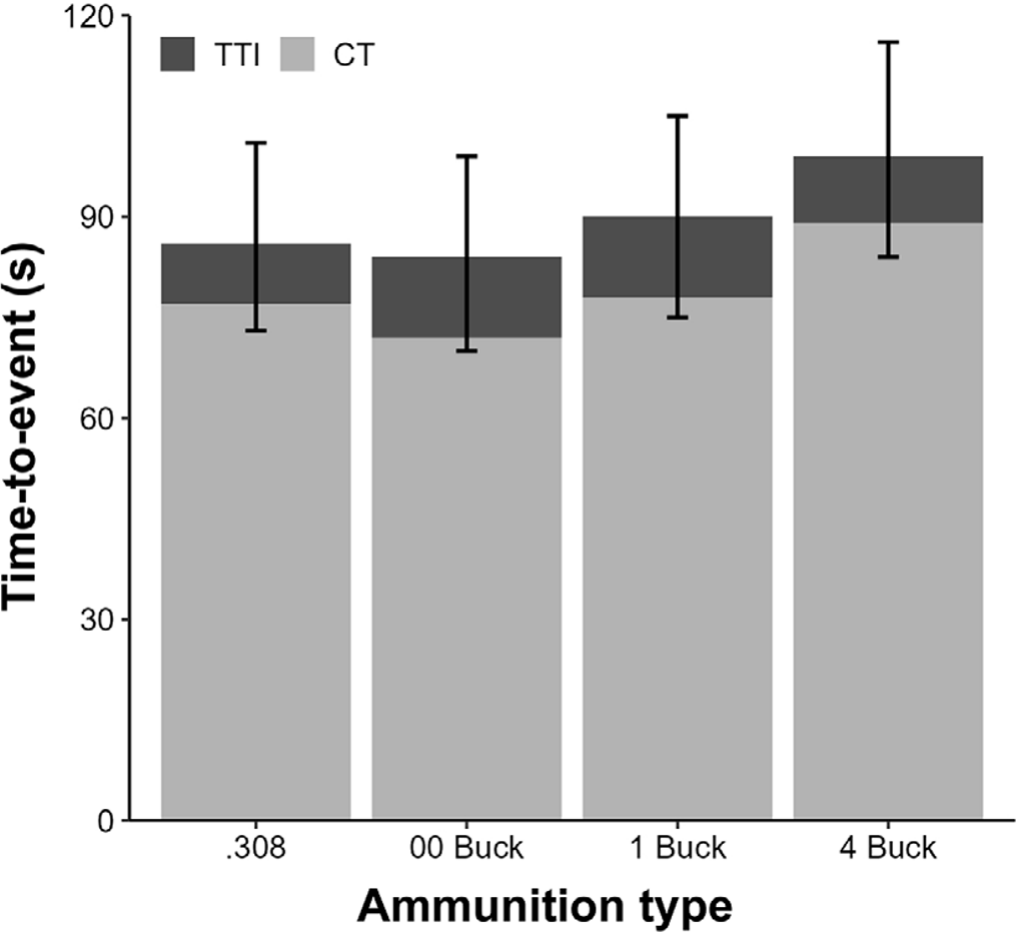
Median chase time (CT), time to insensibility (TTI) and total time (CT + TTI) for fallow deer (*Dama dama*) subjected to aerial culling with one of four ammunition types. Error bars are 90% highest posterior density intervals for TT.

Time-to-event curves for CT, TTI and TT were similar among the four ammunition types (Figure 3). The survival model with no covariate for ammunition type was more parsimonious (DIC = 4,573) and provided a better fit to the data than the model that included ammunition type (DIC = 4,578). CT across all observations ranged from 8 s to 9 min and 47 s. Estimated TTI across all observations ranged from 1 s to 2 min and 4 s. Across all ammunition types, 95% of deer were killed within 43 s of the first shot being fired at them. This period was lowest for deer shot with the .308 rifle (35 s) and ranged from 40 to 47 s for the three shotgun ammunition types. Estimated TT ranged from 14 s to 9 min and 56 s. Frequency distributions for CT, TTI and TT were positively skewed. Median CT, TTI and TT were, however, similar for all four ammunition types (Figure 4; Table A in Supplementary material S4).

Across all ammunition types, the median time between a deer being proclaimed dead by the pilot and the veterinarian conducting the *in situ* post mortem examination was 2 min and 34 s (n = 76; IQR = 2 min 22 s). There was no clear difference in this interval among the ammunition types: the lowest median time to *in situ* post mortem was 2 min and 14 s (.308; IQR = 1 min 30 s), and the greatest median time was 3 min and 28 s (4 Buck; IQR = 1 min 36 s). All 76 deer were assessed as insensible (i.e. absence of a corneal reflex) and dead (i.e. absence of a heartbeat) by the independent veterinarians.

### Non-fatal wounding rate

Since no deer were observed to be either wounded and mobile or wounded and recumbent, the non-fatal wounding rate was 0.00.

### Shots fired and wound tract numbers and locations

The maximum number of shots fired at any of the 390 deer was 14 (from the .308 rifle). This deer ran downhill in steep and densely treed terrain, was hit and fell and became stationary with its thorax partly obscured by understorey vegetation. Several more shots were therefore fired into the visible part of the thorax to ensure that the animal was dead. The mean number of shots fired was similar across all four ammunition types, and the mean number of wound tracts per deer increased across the four ammunition types with the number of projectiles per shot (Table 3). For the three shotgun ammunition types, the number of pellets counted in a deer increased with the number of pellets per shot type (Table 3).

**Table 3.** Mean (± SD) number of shots fired at fallow deer (*Dama dama*) counted during ante mortem observations and mean (± SD) number of wound tracts and shotgun pellets detected during post mortem observations of a subset of shot deer

Firearm/ammunition	Pellets per shot	N ante mortem	Shots fired	N post mortem	Wound tracts	Pellets
.308 rifle	1	99	4.17 (± 2.09)	16	2.2 (± 0.86)	N/A
00 Buck	9	94	4.02 (± 1.82)	9	16.0 (± 6.96)	7.22 (± 4.68)
1 Buck	16	97	3.44 (± 1.80)	19	22.0 (± 10.71)	14.63 (± 9.11)
4 Buck	27	100	3.58 (± 1.73)	15	25.0 (± 7.69)	15.47 (± 6.31)

N: number of deer (sample size). N/A: not available as n = 1

**Figure 5.**
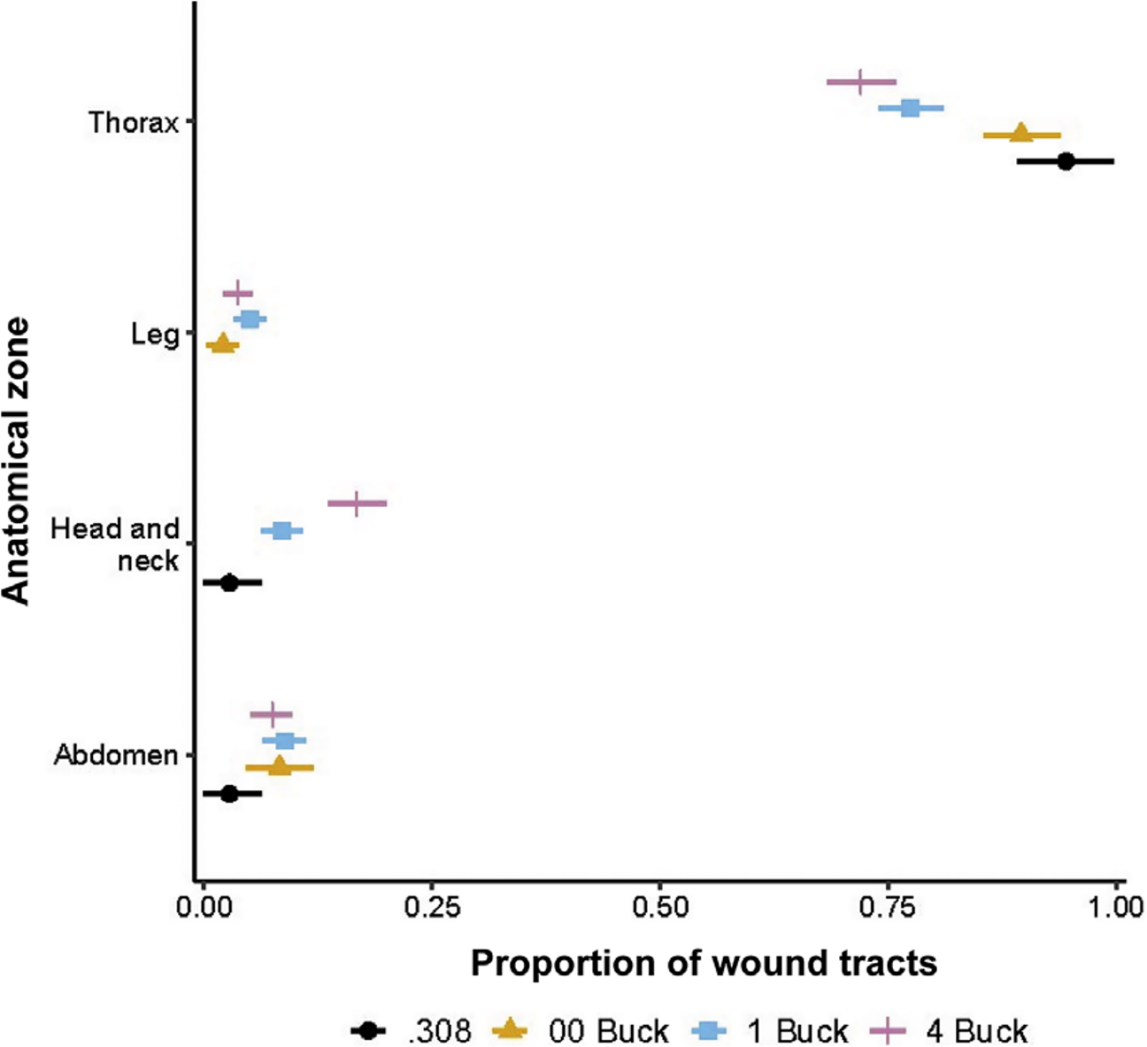
Proportion of wound tracts in four anatomical zones of fallow deer (*Dama dama*) shot with one of four ammunition types during aerial culling in New South Wales, Australia. Horizontal bars indicate 90% HPDIs.

**Figure 6.**
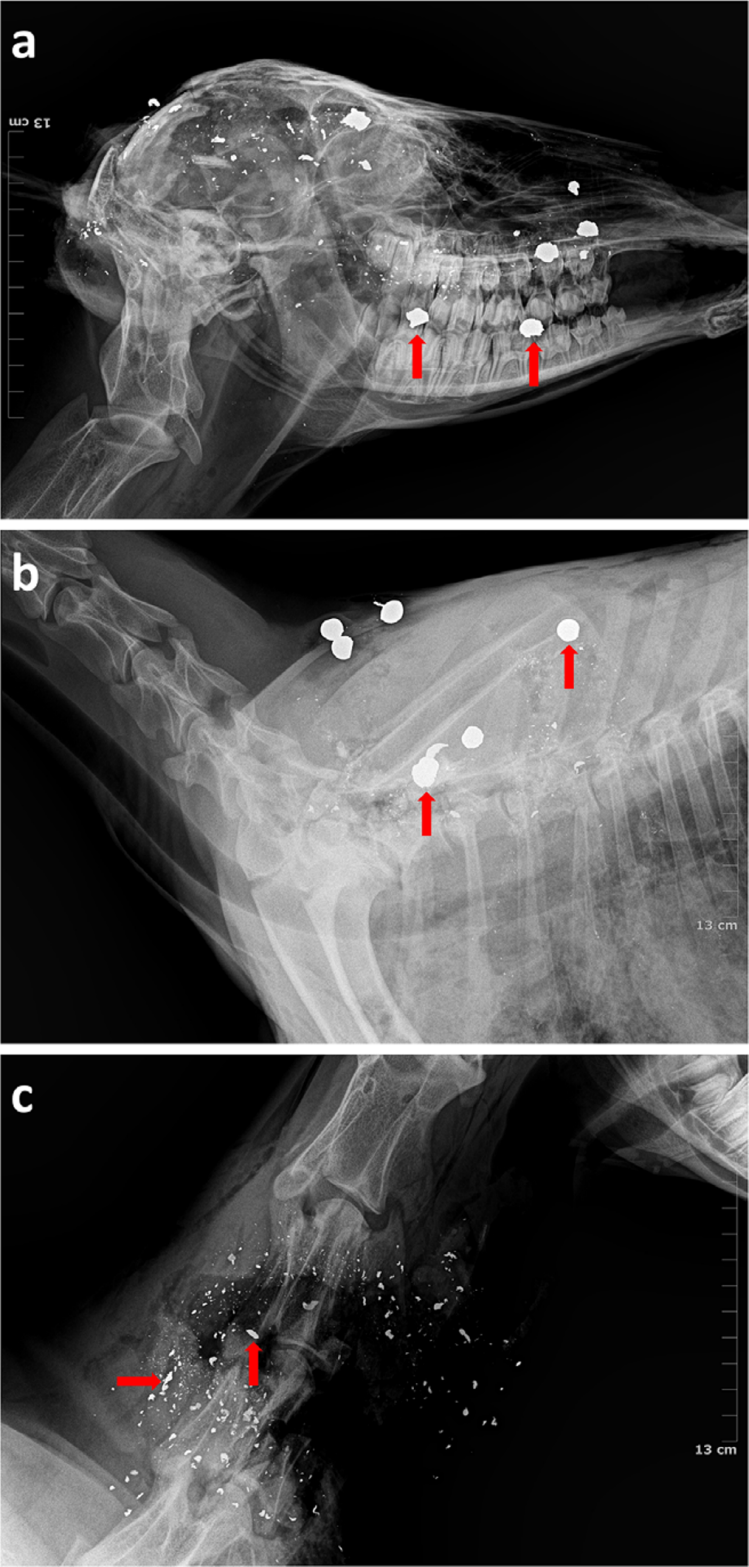
Radiographic images showing locations of 12-gauge shotgun lead pellets and .308 rifle 135-grain lead bullets in fallow deer (*Dama dama*) shot from a helicopter for (a) 1 Buck shotgun pellets in the cranium of an adult female (lateral view), (b) 1 Buck shotgun pellets in the thorax of a juvenile female (lateral view) and (c) 135-grain bullet fragments in the neck of a yearling male (lateral view). Red arrows indicate shotgun pellets (a and b) and bullet fragments (c).

**Figure 7.**
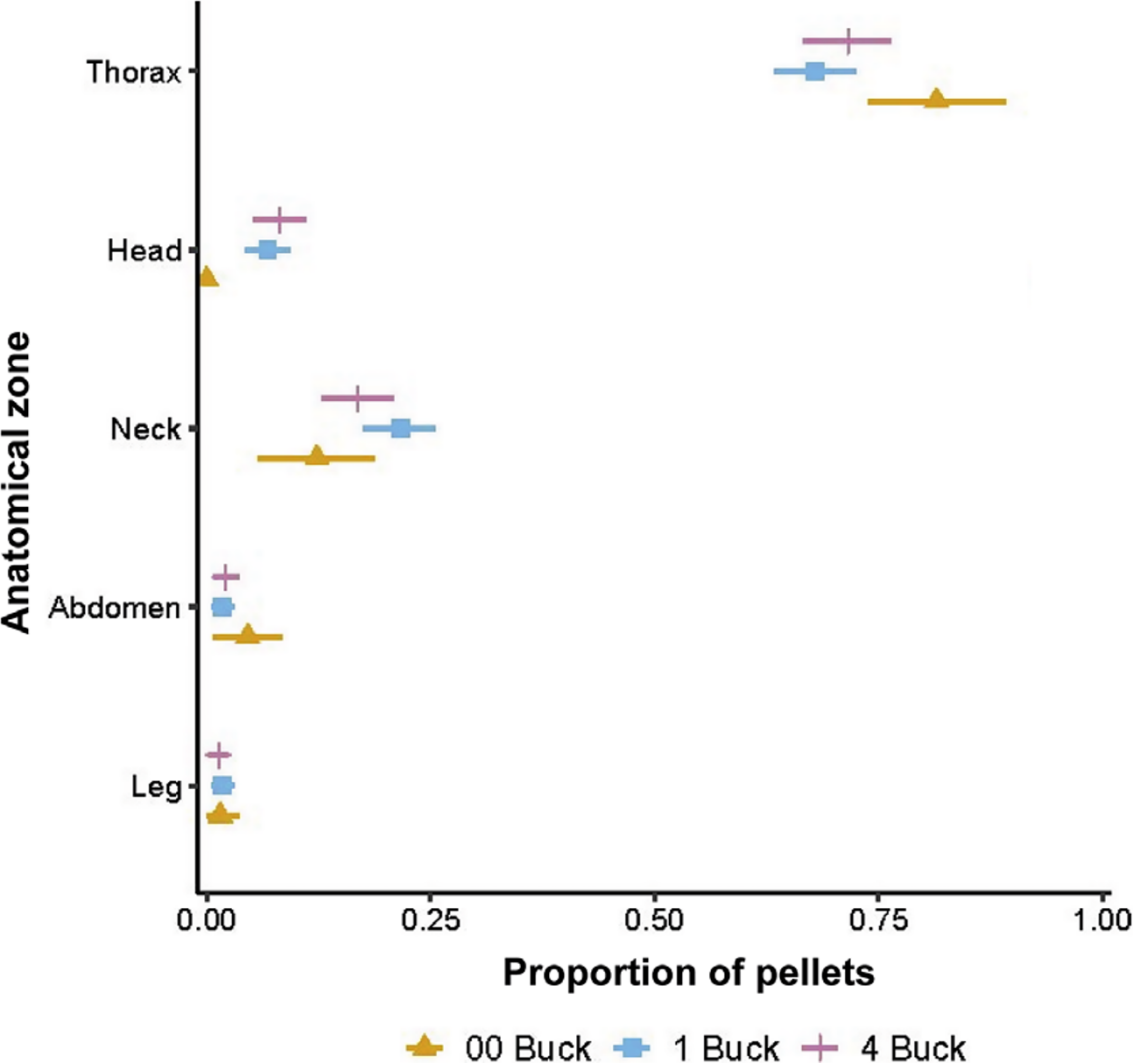
Proportion of shotgun pellets in five anatomical zones of fallow deer (*Dama dama*) shot with one of three shotgun ammunition types from a helicopter in New South Wales, Australia. Horizontal bars indicate 90% HDPIs.

All 59 deer subjected to *ex situ* post mortem examination had ≥ 1 wound tract in the thorax, but the concentration of thoracic wound tracts decreased as the number of projectiles per shot increased (Figure 5). Similarly, all 43 deer that were shot with a shotgun and for which radiographic imagery was available (Figure 6) had ≥ 1 pellet in the thorax, and the greatest concentration of pellets in the thorax was associated with the ammunition type with the fewest pellets (00 Buck; Figure 7). On average, 78% of shotgun pellets fired at deer could not be found in radiographic images and were assumed to have missed or passed through the target (90% HPDI = 77, 80%). The proportion of missing pellets was greater than expected for 4 Buck ammunition ( 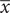 = 0.82, 90% HPDI = 0.80, 0.84) and less than expected for 1 Buck ammunition ( 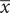 = 0.74, 90% HPDI = 0.71, 0.76).

## Discussion

Wild populations of many non-native deer and other ungulate species are increasing in range and abundance in Australasia (King & Forsyth 2021; Cunningham *et al*. 2022), and aerial culling is a key control tool (Bengsen *et al*. 2022). Continued social licence for aerial culling of ungulates requires that adverse animal welfare outcomes are minimised (Hampton *et al*. 2021a). Our randomised field study revealed that TT (i.e. the duration of stress measured from initiation of pursuit to insensibility), a critical animal welfare parameter for aerial culling, was similar for fallow deer shot with a .308 rifle with 135-grain lead bullets or with a semi-automatic 12-gauge shotgun with three types of buckshot. Importantly, non-fatal wounding was not observed for any of the four ammunition types.

In South Australia, a recent study compared two types of buckshot (GB SG 21-pellet buckshot and Winchester® Super-X 16-pellet buckshot) for aerial shooting of fallow deer (Bradshaw *et al*. 2023). The shooters “*did not observe any difference in performance*” (Bradshaw *et al*. 2023: p 112). The investigators relied upon historical controls (i.e. CT estimates from different target species and environments) when making comparisons between the data they generated and rifle-based shooting. However, they reported a lower average CT associated with the use of a shotgun when compared with historical control data from semi-automatic rifles and inferred that this was evidence that the use of a shotgun in this context makes “*strong improvements to animal welfare*” (Bradshaw *et al*. 2023; p 110). However, after controlling for the influence of helicopter type, pilot, shooter, terrain, and deer density, we found no such evidence in our study. Unquantified differences between the two study areas in concealing cover (a key determinant of the vulnerability of deer to aerial shooting; Nugent *et al*. 1987) could have contributed to the different results. In our study area, adult female fallow deer fitted with GPS-tracking collars used treed areas more during day-time (when the aerial culling was conducted) compared to night-time (Bengsen *et al*. 2024). The dissimilarities between our findings and those of Bradshaw *et al.* (2023) also emphasise the importance of adequate sample sizes (Hampton *et al*. 2019) and randomisation when comparing treatment groups with the control (Ryeng & Larsen 2021).

Previous studies of the animal welfare outcomes of aerial culling have been observations of ‘routine’ control programmes. We attempted to incorporate the key elements of robust experimental design in our field study: one or more treatments and a control; conducting a sample size calculation to determine how many individuals should be included in the treatments and control groups; randomly assigning the treatments and control to sorties; and analysis focusing on the research question that led to the trial (Fisher 1947; Hawkins *et al*. 2013). In particular, we considered *a priori* that deer density (which declines as animals are removed during the aerial culling programme (Bengsen *et al*. 2022) and concealing cover (Hampton *et al*. 2021a) could potentially affect animal welfare outcomes. Future studies of the animal welfare outcomes of lethal control tools would benefit from following, as far as is practical, the key features of robust experimental design. Given that concealing cover is a key determinant of the vulnerability of deer to being shot from a helicopter (Nugent *et al*. 1987), future studies could consider comparing areas with large differences in the extent of forest vs grassland.

For consistency among treatments and sorties, our study used one shooter. There can be variation among individual shooters in the animal welfare outcomes of aerial culling of ungulates (Hampton *et al*. 2014, 2017). Our study used one of the longest-serving shooters in NSW. Other shooters, particularly those with substantially less experience with the firearm–ammunition combinations used in our trial, might have produced different animal welfare outcomes from those reported here.

Our trial was conducted approximately four weeks prior to the peak of the rut, and many yearling and adult males were sexually segregated from the females and juveniles (Chapman & Chapman 1997). Our sample of fallow deer was biased towards adult males because during this study that age-sex class occupied more open areas (i.e. less tree cover) than did adult females (Bengsen *et al*. 2024). Hence, adult males were more likely to be detected and shot, particularly early in the trial. This pattern validates our decision to randomly assign ammunition types, without replacement, to sorties. Adult males were also near the peak of their annual body mass cycle (Mulley 2007), with seven exceeding 100 kg. In New Zealand, aerial cullers prefer to use the .308 rifle to shoot large adult male red deer, because it is believed that Winchester 9 pellet 00 Buck (the preferred shotgun ammunition) pellets do not always penetrate the cranium, given that it is hit (M Beardsley, Department of Conservation, unpublished data). Further work is needed to identify the limitations, if any, of using buckshot ammunition for deer > 100 kg.

In our study, 95% of fallow deer were killed within 43 s of the first shot being fired at them. This is almost identical (95% of deer killed within 42 s) to that observed in a previous study of the animal welfare outcomes of aerial culling of fallow deer using the .308 rifle and 135-grain lead bullets conducted ~100 km south-west of our study area (Hampton *et al*. 2021a). Given that the same helicopter and shooter was used (but a different pilot), it is perhaps unsurprising that similar TTs were observed for the .308 rifle with 135-grain ammunition in that observational study and in this randomised field study.

The recommended effective range of the 12-gauge shotgun (25–30 m) (Feral Animal Aerial Shooting Team 2020) is much less than that of the .308 rifle (> 200 m) (Hampton *et al*. 2022). Maximum effective range recommendations aside, the onus is on the shooter to take shots at distances and in situations that minimise potential animal suffering. This responsibility is sometimes referred to as the ‘ethical limit’ (Caudell *et al*. 2009) or ‘ethical range’ (Caudell *et al*. 2012) for any firearm–ammunition–target species combination. Hence, the ‘ethical range’ is likely to be less than those maximum effective range distances and will likely vary among shooters depending on factors including their experience with the firearm–ammunition combination, the target species, and the habitat they are encountered in. We attempted to record the shooting distances for shots fired in our trial, but this proved impractical because of the absence of laser range finders in standard aerial culling methods and the need for a shooter and observer to focus on the fate of live animals during culling programmes. In tall timber, shooting distances can greatly exceed 30 m, so the .308 rifle would be needed to shoot fallow deer in this habitat type.

A large proportion of pellets fired at deer were not detected in X-rays. The undetected pellets would include those that: (i) missed the deer; (ii) passed through the deer but did not embed; and (iii) entered the deer and fragmented to such an extent that they could not be enumerated as pellets (Green *et al.* 2022). For relatively small animals shot using shotguns (e.g. waterfowl), the proportion of pellets fired from a cartridge that are found embedded in tissues is relatively low, with the majority of pellets either missing or passing through the body (Noer *et al*. 2007). Several recent studies have reported the number of embedded pellets detected via X-ray in ungulates killed with shotguns (Wilson *et al*. 2020; Nkosi *et al*. 2022). A USA study showed that lead fragments are often found in the meat of white-tailed deer (*Odocoileus virginianus*) harvested by recreational hunters using shotguns (Wilson *et al*. 2020), indicating that fragmentation of lead shotgun pellets in deer is common.

It is likely that the use of lead-based projectiles (shot and bullets) in our study would have affected the frequency with which projectiles were retained within carcases and visible in X-rays. Lead-free shotgun ammunition is increasingly being used in wildlife management, for example, for waterfowl hunting globally (Kraabel *et al.* 1996), or for the aerial shooting of Himalayan tahr (*Hemitragus jemlahicus*) in New Zealand (Buenz *et al.* 2024). The properties of non-lead shotgun pellets are likely to be considerably different to those of lead-based shot. Steel, for example, is much less prone to deformation and fragmentation, and may penetrate tissues differently to lead, altering the likelihood of pellets passing through an animal (‘through-body strikes’; Pierce *et al.* 2015). Our results support the growing body of international evidence demonstrating that contamination of deer carcases occurs whenever lead-based ammunition is used, regardless of whether it is derived from rifle bullets (Hampton *et al*. 2023) or shotgun pellets (Wilson *et al*. 2020). We suggest that future studies might investigate the use of lead-free shot for the aerial culling of deer.

Non-fatal wounding was not observed in our trial. This is an important finding because we implemented the most rigorous methodology yet reported for assessing the occurrence of non-fatal wounding in aerial culling. An innovation of our study was to land as soon as possible and as close as possible to immobile deer and test whether they were insensible and dead. Due to most deer being shot in steep and/or densely treed terrain, we could safely conduct this assessment for only 76 (20%) of the shot deer. However, all 76 deer were confirmed dead. Importantly, these checks were performed at a median of 2 min 34 s (IQR = 2 min 22 s) after the inspected deer was deemed to be insensible by the observer. It is unlikely that animals could be inspected more quickly in an aerial culling programme, given the need to safely land the helicopter close to a shot animal, and then safely exit the helicopter and locate the shot animal. Our *in situ* post mortem results suggest that the deer for which we could not perform post mortem inspections were all likely to have been dead. More generally, this finding indicates that ante mortem observations of immobile shot animals (i.e. as used in all previous studies) usually equates to death. However, this finding does not indicate that non-fatal wounding never occurs in aerial culling of deer under the culling protocols used in this study, only that it occurs at a sufficiently low frequency that it was not detected in our sample. We emphasise that our sample is from one shooting team (i.e. one shooter and one pilot) that knew they were being assessed on the frequency of non-fatal wounding.

The methods used in this study could be refined to improve the quality of the data collected. In particular, the position of the observer in the helicopter meant that the proportion of events that could not be observed and for which TTI was censored was ~23% across the four ammunition types. Our estimates of the duration of stress could have been biased if the sample of observed animals somehow differed from the unobserved animals. One solution would be to mount a video camera behind and above the shooter (inside the helicopter), or on the helmet of the shooter, as has been used to estimate time-to-event data in helicopter-based wildlife capture studies (Latham *et al*. 2019). However, this was not possible in the present study due to procedural restrictions on recording images of aerial culling (Feral Animal Aerial Shooting Team 2020).

### Animal welfare implications

The animal welfare outcomes of aerial culling of non-native fallow deer were similar for the three shotgun ammunition types and the .308 rifle ammunition. The implication is that using a .308 semi-automatic rifle with 135-grain ammunition or a 12-gauge semi-automatic shotgun with Winchester^®^ 00 Buck, 1 Buck or 4 Buck ammunition would have little effect on the animal welfare outcomes of aerial culling of fallow deer under the protocols adhered to in this study, provided that the shorter range limit of the shotgun (~30 m) is observed. If two firearms were carried in the helicopter, then one could be the shotgun (for shooting at deer ≤ 100 kg and ≤ 30 m) and the other the .308 (for shooting at deer > 100 kg and > 30 m).

## Supporting information

Forsyth et al. supplementary material Forsyth et al. supplementary material

## Supplementary material

The supplementary material for this article can be found at http://doi.org/10.1017/awf.2025.10037.
