# Supplementary material for: Rifles and shotguns have similar animal welfare outcomes during aerial culling of non-native fallow deer (Dama dama)
Source: Anim Welf. 2025 Sep 18;34:e63. doi: 10.1017/awf.2025.10037 (PMC12451392; doi:10.1017/awf.2025.10037)
Supplement: Forsyth et al. supplementary material [file S0962728625100377sup001.zip › Supplementary material S1.pdf]

Supplementary material S1. Fallow deer population density estimation

Rifles and shotguns have similar animal welfare outcomes during aerial culling of non-native fallow deer (*Dama dama*)

David M Forsyth[1,2], Andrew J Bengsen[3], Andrew L Perry[4], Lee Parker[3], Mal Leeson[5], Jordan O Hamptonhttps://orcid.org/0000-0003-0472-3241[6,7]

[1]Vertebrate Pest Research Unit, NSW Department of Primary Industries and Regional Development, Orange, NSW, Australia
[2]School of Biological, Earth & Environmental Sciences, University of New South Wales, Sydney, NSW, Australia
[3]Vertebrate Pest Research Unit, NSW Department of Primary Industries and Regional Development, Calala, NSW, Australia
[4]Ecotone Wildlife Veterinary Services, Inverloch, VIC, Australia
[5]Central Tablelands Local Land Services, Mudgee, NSW, Australia
[6]School of Veterinary Medicine, Murdoch University, Murdoch, WA, Australia
[7]Faculty of Science, University of Melbourne, Parkville, VIC, Australia
Author for correspondence: Jordan O Hampton, email: jordan.hampton@murdoch.edu.au

Field methods

The density of fallow deer in our study area was estimated in the week preceding our trial by visual survey from a Bell 206 Jet Ranger helicopter, following the method reported for this area (site ‘NSW4’) (Bengsen *et al*. 2022). Briefly, the helicopter was flown at ~46 m above ground level at 85 km per hour in the 2 hours after sunrise and before sunset, when deer are most active. We used an equal-spaced zigzag transect design to maximise survey effort while minimising variability in coverage probability and bias. Transects were flown three times each, but never on the same day, so that any deer that were disturbed on or near the transect lines by the previous survey would have redistributed themselves. One observer was seated in

the front of the helicopter, adjacent to the pilot, and another observer was seated behind the first observer on the same side. Sighting booms with five distance classes (0–20 m; 20–40 m; 40–70 m; 70–100 m; 100–150 m) were fitted for each observer and calibrated using markers set at known distances on the ground prior to undertaking surveys. Each observer's observations were independently georeferenced and recorded using individual USB game controllers and GPS receivers providing input to a customised app (McLeod 2018) running on a detachable screen notebook (Bengsen *et al*. 2022). The species, group size, distance class, habitat (open or wooded) and geographic coordinates of the helicopter were recorded for each observation. In addition to fallow deer (*Dama dama*), red deer (*Cervus elaphus*) and rusa deer (*Cervus timorensis*) were also observed in our study area, but there were too few detections for estimating their density. The aerial surveys were conducted with approval from the New South Wales Department of Primary Industries' Orange Animal Ethics Committee (Approval number OAEC-0270 (ORA 20/23/001)).

Statistical analysis

The fallow deer aerial survey data were analysed using mark–recapture distance sampling (MRDS) (Burt *et al*. 2014; Peterson *et al*. 2020) using the method described for this area in reference (Bengsen *et al*. 2022). Briefly, models included a mark–recapture submodel for estimating g(0), the probability of detecting deer on the transect line, and a multi-covariate distance sampling (MCDS) submodel for estimating the distance sampling detection function using observation-specific covariates, including habitat (open or wooded), distance class, observer position (front or rear) and deer group size. Independent deer group detections were compared between observers by using GPS location, group size, and distance class to create a detection history for each group, as follows: 10 if the group was detected by the front observer only, 01 if detected by the rear observer only, or 11 if detected by both observers. We fitted independent observer models to the detection dataset using a two-stage process.

[OFFICIAL]

First, we fitted a series of null models for different distance sampling detection functions (hazard rate and half-normal with cosine and simple polynomial adjustments) and selected the best-supported detection function using Akaike's information criterion (AIC) (Burnham & Anderson 2002). We then used the best-supported detection function to fit a series of MCDS submodels with combinations of covariates and selected the best-supported model using AIC. Goodness-of-fit was assessed by examining tables of observed and predicted detections. Models were fitted using the MRDS package (Laake *et al*. 2020) in the R statistical environment (R Core Team 2022).

Results

A total of 497 groups of fallow deer were detected by either one or both of the two observers. Group sizes ranged from 1 to 20, with a mean of 2.9. A distance model using a hazard rate key function with deer group size as a detection covariate provided the best fit to the data. The estimated fallow deer density was 37.5 deer per km^2 (95% CI: 25.3–55.8).

References

Bengsen AJ, Forsyth DM, Pople A, Brennan M, Amos M, Leeson M, Cox TE, Gray B, Orgill O, Hampton JO, Crittle T and Haebich K 2022 Effectiveness and costs of helicopter-based shooting of deer. *Wildlife Research* **50**(9): 617–631.

Burnham KP and Anderson DR 2002 *Model selection and multimodel inference: a practical information–theoretic approach*. *Second Edition.* Springer-Verlag, New York, USA.

Burt ML, Borchers DL, Jenkins KJ and Marques TA 2014 Using mark–recapture distance sampling methods on line transect surveys. *Methods in Ecology and Evolution* **5**(11): 1180–1191.

Laake J, Borchers D, Thomas L, Miller D and Bishop J 2020 *mrds: mark–recapture distance sampling. R package version 2.4.4*. https://CRAN.R-project.org/package=mrds (accessed 3 July 2023).

McLeod SR 2018 *Deer aerial survey logger. v1.0.* Vertebrate Pest Research Unit, NSW Department of Primary Industries: Orange, Australia.

Peterson MK, Foley AM, Tri AN, Hewitt DG, DeYoung RW, DeYoung CA and Campbell TA 2020 Mark–recapture distance sampling for aerial surveys of ungulates on rangelands. *Wildlife Society Bulletin* **44**(4): 713–723.

R Core Team 2022 *R: a language and environment for statistical computing*. https://www.R-project.org (accessed 8 July 2024).

[OFFICIAL]
