# Supplementary material for: Rifles and shotguns have similar animal welfare outcomes during aerial culling of non-native fallow deer (Dama dama)
Source: Anim Welf. 2025 Sep 18;34:e63. doi: 10.1017/awf.2025.10037 (PMC12451392; doi:10.1017/awf.2025.10037)
Supplement: Forsyth et al. supplementary material [file S0962728625100377sup001.zip › Supplementary material S2.pdf]

Supplementary material S2. Sample size calculation

Rifles and shotguns have similar animal welfare outcomes during aerial culling of non-native fallow deer (*Dama dama*)

David M Forsyth[1,2], Andrew J Bengsen[3], Andrew L Perry[4], Lee Parker[3], Mal Leeson[5], Jordan O Hamptonhttps://orcid.org/0000-0003-0472-3241[6,7]

[1]Vertebrate Pest Research Unit, NSW Department of Primary Industries and Regional Development, Orange, NSW, Australia
[2]School of Biological, Earth & Environmental Sciences, University of New South Wales, Sydney, NSW, Australia
[3]Vertebrate Pest Research Unit, NSW Department of Primary Industries and Regional Development, Calala, NSW, Australia
[4]Ecotone Wildlife Veterinary Services, Inverloch, VIC, Australia
[5]Central Tablelands Local Land Services, Mudgee, NSW, Australia
[6]School of Veterinary Medicine, Murdoch University, Murdoch, WA, Australia
[7]Faculty of Science, University of Melbourne, Parkville, VIC, Australia
Author for correspondence: Jordan O Hampton, email: jordan.hampton@murdoch.edu.au

We wanted to estimate the extent to which each of the three shotgun treatments was associated with an increase in the expected total time from starting a pursuit to an animal's death or insensibility (as inferred from remote observation), relative to using a .308 rifle with 135-grain bullets. We were specifically interested in detecting increases in total time, rather than decreases, because that would indicate an increase in the duration of stress prior to animals being killed. There are no established guidelines or standards for what constitutes an acceptable total time for aerial culling, but we consider that an increase of >50% of the expected duration compared with what has been observed for the .308 rifle with 135-grain bullets would be a strong indication that the shotgun caused unnecessary increased stress for fallow deer (*Dama dama*) subject to aerial culling.

[OFFICIAL]

Previously estimated total times using the .308 rifle with 135-grain bullets were log-normally distributed, with a median of 133 s, a geometric mean of 148 s and 5th and 95th percentiles of 50 and 700 s, respectively (New South Wales data only; Hampton *et al.* 2021). After log transformation, the total time was therefore approximated by a normal distribution with $\text{mean}_{\text{reference}} = 5.003$ and $\text{s.d.}_{\text{reference}} = 0.736$. This provided our reference distribution ($\text{CV}_{\text{reference}} = 0.147$). We used Monte Carlo simulation to assess the power of one-way ANOVA to detect meaningful differences between a range of effect sizes using different sample sizes. There were many possible combinations of outcomes that could be simulated with three treatment groups and one control, but we focused on all treatment groups having a consistently greater total time than the control, because we did not have any prior information to suggest that one treatment should differ from any other. Effect sizes were expressed as increases in total time, relative to the previously estimated expected total time using a .308 rifle. Effect sizes included increases in total time of 37 s (25% increase), 48 s (33% increase), 60 s (40% increase) and 74 s (50% increase). Simulated sample sizes in each of the three treatments and in the control ranged from 40 to 140 individuals. Simulated data (N = 10 000 simulations) were drawn from a log-normal distribution with $\text{mean}_{\text{sim}} = \log[\exp(\text{mean}_{\text{reference}}) + \text{effect size}]$ and $\text{s.d.}_{\text{sim}} = \text{CV}_{\text{reference}} \times \text{mean}_{\text{sim}}$. Power was assessed as the proportion of ANOVAs that identified evidence against the null hypothesis at $\alpha = 0.05$. Our simulations (Table A) indicated that a sample size of 100 animals per group would provide a 78% probability of detecting an increase of 48 s, or 33% relative to data reported for the .308 rifle with 135-grain bullets. The same sample size would provide 91% and 98% power for effect sizes of 60 and 74 s, respectively (Table A), under the assumed treatment effects. We therefore propose that a sample size of approximately 100 fallow deer in each of the three treatments and in the control group is required to make meaningful conclusions about total times.

Table A. Results of ANOVA to test for differences between a single simulated treatment group and the reference data.

N	Effect size (s)			
	37	48	60	74
40	0.229	0.365	0.488	0.656
60	0.342	0.532	0.700	0.852
80	0.450	0.675	0.828	0.941
100	0.551	0.778	0.911	0.980
120	0.639	0.859	0.956	0.995
140	0.721	0.905	0.978	0.998

Values in columns 2–5 represent the estimated power of the test to reject the null hypothesis at $P < 0.05$. Effect size (s) represents the difference between the simulated treatment and the reference data, and N (column 1) is the simulated sample size per group.

Reference

Hampton JO, Bengsen AJ, Pople A, Brennan M, Leeson M and Forsyth DM 2021 Animal welfare outcomes of helicopter-based shooting of deer in Australia. *Wildlife Research* **49**(3): 264–273. https://doi.org/10.1071/WR21069
