# Supplementary material for: Rifles and shotguns have similar animal welfare outcomes during aerial culling of non-native fallow deer (Dama dama)
Source: Anim Welf. 2025 Sep 18;34:e63. doi: 10.1017/awf.2025.10037 (PMC12451392; doi:10.1017/awf.2025.10037)
Supplement: Forsyth et al. supplementary material [file S0962728625100377sup001.zip › Supplementary material S3.pdf]

Supplementary material S3. Order of ammunition type and the number of fallow deer (*Dama dama*) assessed in each of the sorties

Rifles and shotguns have similar animal welfare outcomes during aerial culling of non-native fallow deer (*Dama dama*)

David M Forsyth[1,2], Andrew J Bengsen[3], Andrew L Perry[4], Lee Parker[3], Mal Leeson[5], Jordan O Hamptonhttps://orcid.org/0000-0003-0472-3241[6,7]

[1]Vertebrate Pest Research Unit, NSW Department of Primary Industries and Regional Development, Orange, NSW, Australia
[2]School of Biological, Earth & Environmental Sciences, University of New South Wales, Sydney, NSW, Australia
[3]Vertebrate Pest Research Unit, NSW Department of Primary Industries and Regional Development, Calala, NSW, Australia
[4]Ecotone Wildlife Veterinary Services, Inverloch, VIC, Australia
[5]Central Tablelands Local Land Services, Mudgee, NSW, Australia
[6]School of Veterinary Medicine, Murdoch University, Murdoch, WA, Australia
[7]Faculty of Science, University of Melbourne, Parkville, VIC, Australia
Author for correspondence: Jordan O Hampton, email: jordan.hampton@murdoch.edu.au

We included only one of the four ammunition types within a sortie because of the risk of mixing shotgun ammunition types. Based on our previous experience of aerial culling of fallow deer (*Dama dama*) in this area and elsewhere (Bengsen *et al*. 2023), we suspected that at the beginning of the trial (i.e. earlier sorties) deer would be detected and shot in the flatter and sparsely treed cropped and pastoral areas that surrounded the staging area, and that as these deer were progressively removed the culling team would increasingly need to travel further from the staging area and into steeper and more densely treed terrain to detect and shoot fallow deer. Therefore, to minimise the potential effects of terrain and vegetation type on the results of this trial, we randomly allocated, using a random number generator, each of

the four ammunition types to sorties without replacement until all four ammunition types were represented, and then the process was repeated (Table A). To further explain this, consider sorties 1–4 in Table A. The first randomly selected sortie was Shotgun 1 Buck, leaving three possibilities for sortie 2 (Shotgun 00 Buck, Shotgun 4 Buck and Rifle 135-grain). Shotgun 4 Buck was randomly selected from those three possibilities, leaving two possibilities for sortie 3 (Shotgun 00 Buck and Rifle 135-grain). Rifle 135-grain was randomly selected from those two possibilities and hence sortie 4 had to be Shotgun 4 Buck. The same process was followed for sorties 5–8, 9–12, 13–16 and 17–20 (but only 17 sorties were needed; Table A). The independent veterinarian kept a running tally of the number of fallow deer shot for each of the four ammunition types. Sample sizes were slightly less than the desired 100 for three of the four ammunition types (Shotgun 00 Buck, Shotgun 1 Buck and Rifle 135-grain) due to tallying errors.

In the first three sorties, fallow deer were detected and shot on the open cropping paddocks and sparsely treed pasture paddocks near to the helicopter staging area (Figures A and B). The majority of these deer were adult males, often in groups of up to 22 individuals (Figures A and B). As the trial progressed and these deer were shot and removed from the population, the culling team had to travel increasingly further from the staging area and into steeper and more densely treed areas within the culling zone to detect and shoot fallow deer (Figure C); in these habitats, mostly female and juvenile fallow deer were encountered (Bengsen *et al*. 2024). It was often not safe to land the helicopter and conduct *in situ* post-mortem assessments for deer shot in later sorties. Hence, both the absolute number and the proportion of fallow deer (compared with those assessed ante-mortem) declined as the study progressed (Table A).

References

Bengsen AJ, Comte S, Parker L, Forsyth DM and Hampton JO 2024 Site fidelity trumps disturbance: aerial shooting does not cause surviving fallow deer (*Dama dama*) to disperse. *Wildlife Research* **51**(9): WR24098. https://doi.org/10.1071/WR24098

Bengsen AJ, Forsyth DM, Pople A, Brennan M, Amos M, Leeson M, Cox TE, Gray B, Orgill O, Hampton JO, Crittle T and Haebich K 2022 Effectiveness and costs of helicopter-based shooting of deer. *Wildlife Research* **50**(9): 617–631. https://doi.org/10.1071/WR21156

Table A. Order of firearm and ammunition type and the number of fallow deer (*Dama dama*) assessed in each of the 17 sorties.

Sortie	Firearm and ammunition	Date	Start time	End time	Number of fallow deer assessed			
					Ante mortem	TTI censored	Post mortem *in situ*	Post mortem *ex situ*
1	Shotgun 1 Buck (16 pellet)	20/02/2023	0855h	1048h	13	8	10	8
2	Shotgun 4 Buck (27 pellet)	20/02/2023	1142h	1333h	9	3	6	6
3	Rifle 135-grain	20/02/2023	1514h	1659h	27	4	8	8
4	Shotgun 00 Buck (9 pellet)	21/02/2023	0753h	0939h	45	5	8	7
5	Shotgun 4 Buck (27 pellet)	21/02/2023	1033h	1219h	42	13	6	4
6	Rifle 135-grain	21/02/2023	1427h	1536h	29	8	7	4
7	Shotgun 00 Buck (9 pellet)	21/02/2023	1616h	1757h	31	11	6	2
8	Shotgun 1 Buck (16 pellet)	22/02/2023	0752h	0910h	20	0	6	5
9	Shotgun 1 Buck (16 pellet)	22/02/2023	1054h	1122h	41	10	4	4
10	Rifle 135-grain	22/02/2023	1311h	1428h	29	12	3	3
11	Shotgun 4 Buck (27 pellet)	22/02/2023	1531h	1634h	18	0	4	2
12	Shotgun 00 Buck (9 pellet)	23/02/2023	0918h	1051h	13	5	2	0
13[A]	Rifle 135-grain	23/02/2023	1208h	1243h	11	1	1	1
14	Shotgun 4 Buck (27 pellet)	23/02/2023	1339h	1443h	31	1	3	3
15	Shotgun 1 Buck (16 pellet)	23/02/2023	1519h	1620h	23	8	2	2

[OFFICIAL]

16	Shotgun 00 Buck (9 pellet)	23/02/2023	1620h	1624h	5	0	0	0
17	Rifle 135-grain	23/02/2023	1625h	1629h	3	0	0	0

For an explanation of the two firearm and four ammunition types, see the main text. TTI, time to insensibility.

[OFFICIAL]

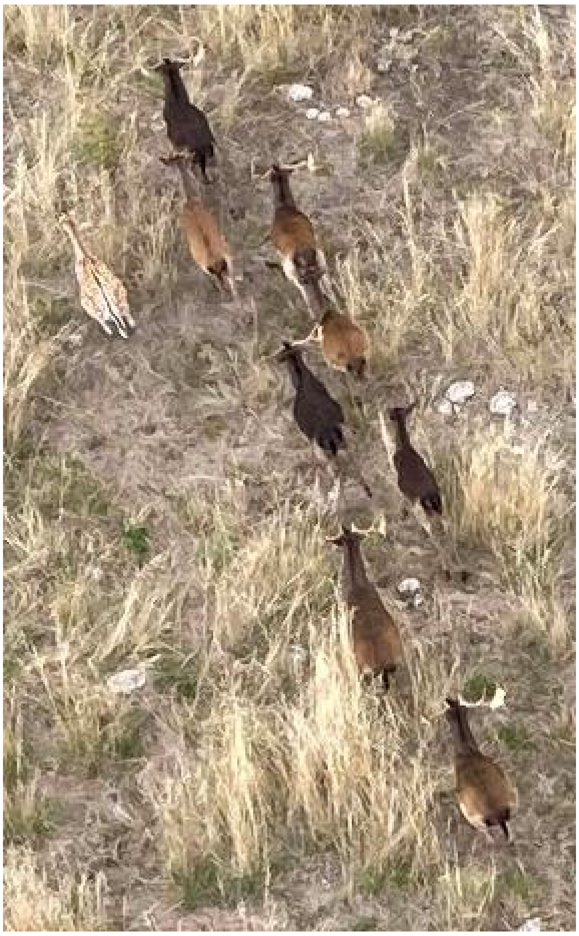

Figure A. A group of nine fallow deer (*Dama dama*), predominantly adult males, encountered during sortie 9 of our trial. Adult male fallow deer were commonly encountered in sparsely treed pasture such as shown here. However, as the trial progressed and these deer were killed, the culling team needed to travel to steeper and more densely treed areas to detect and shoot fallow deer (image: M Leeson).

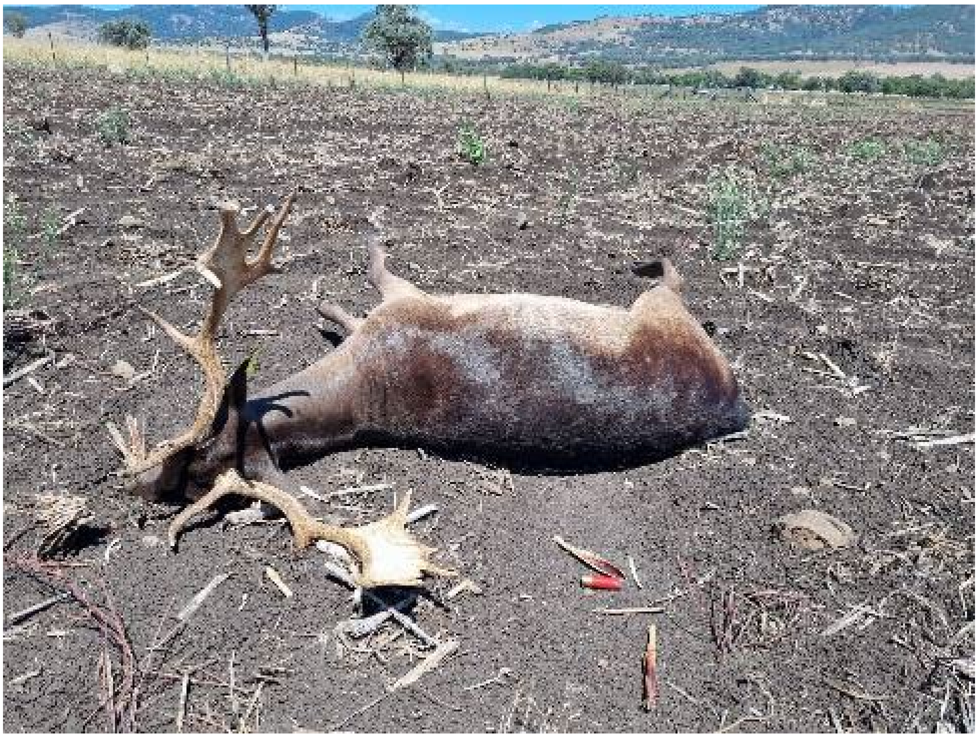

Figure B. An adult male fallow deer (*Dama dama*) shot on a sorghum stubble on sortie 1 in our trial. Note the red shotgun cartridge in the foreground, and the yellow cattle-tag in the deer's right ear. Adult male fallow deer were commonly encountered in these flat and sparsely treed cropping paddocks early in the trial. As the trial progressed and deer living in the flatter and more sparsely treed areas were removed, the culling team had to travel into the steeper and more densely treed areas visible in the far background to detect and shoot fallow deer (image: J Hampton).

[OFFICIAL]

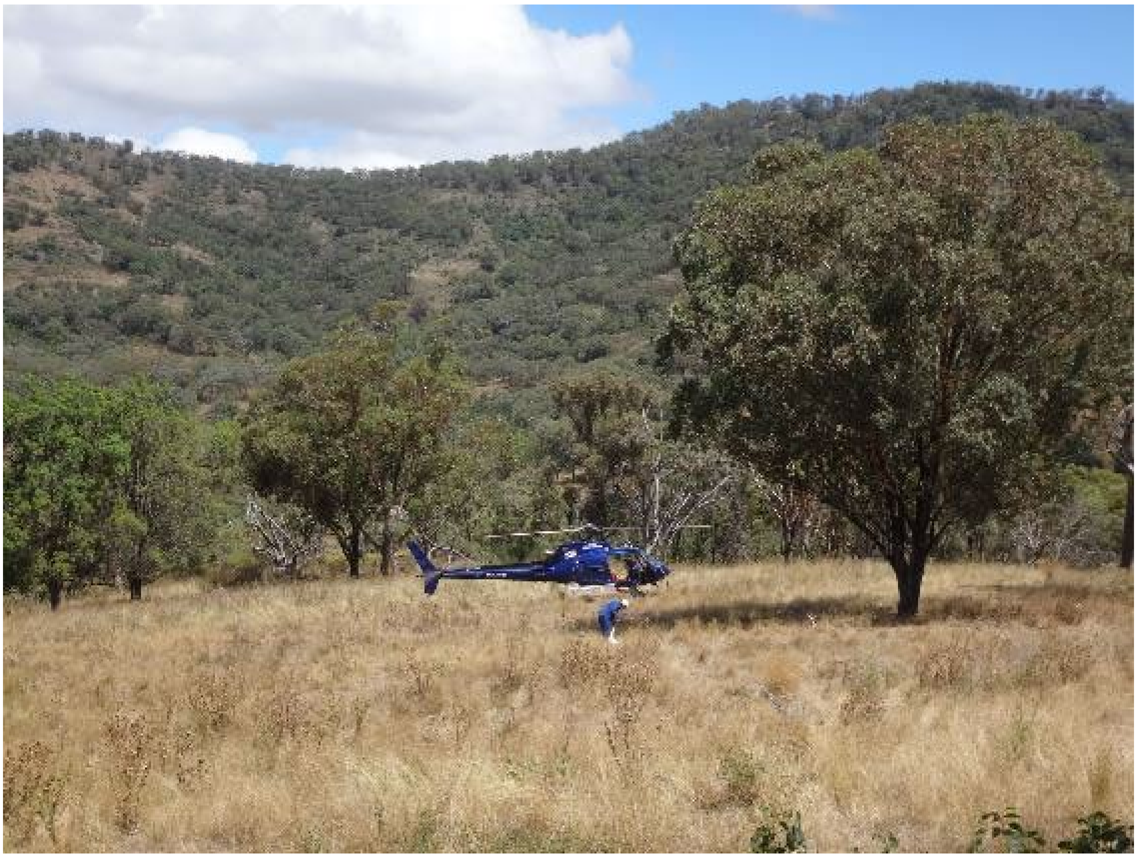

Figure C. As the trial progressed, the culling team needed to hunt the steeper and more densely treed areas, such as shown in the background of this image. Adult female and juvenile fallow deer (*Dama dama*) were mostly encountered in these steeper and more densely treed areas. The steep terrain and dense tall trees in these areas meant that it was often not safe to land the helicopter and conduct post mortem assessments (image: J Hampton).

[OFFICIAL]
