# Supplementary material for: Rifles and shotguns have similar animal welfare outcomes during aerial culling of non-native fallow deer (Dama dama)
Source: Anim Welf. 2025 Sep 18;34:e63. doi: 10.1017/awf.2025.10037 (PMC12451392; doi:10.1017/awf.2025.10037)
Supplement: Forsyth et al. supplementary material [file S0962728625100377sup001.zip › Supplementary material S4.pdf]

Supplementary material S4. Summary statistics for chase time, time to insensibility and total time

Rifles and shotguns have similar animal welfare outcomes during aerial culling of non-native fallow deer (*Dama dama*)

David M Forsyth[1,2], Andrew J Bengsen[3], Andrew L Perry[4], Lee Parker[3], Mal Leeson[5], Jordan O Hamptonhttps://orcid.org/0000-0003-0472-3241[6,7]

[1]Vertebrate Pest Research Unit, NSW Department of Primary Industries and Regional Development, Orange, NSW, Australia
[2]School of Biological, Earth & Environmental Sciences, University of New South Wales, Sydney, NSW, Australia
[3]Vertebrate Pest Research Unit, NSW Department of Primary Industries and Regional Development, Calala, NSW, Australia
[4]Ecotone Wildlife Veterinary Services, Inverloch, VIC, Australia
[5]Central Tablelands Local Land Services, Mudgee, NSW, Australia
[6]School of Veterinary Medicine, Murdoch University, Murdoch, WA, Australia
[7]Faculty of Science, University of Melbourne, Parkville, VIC, Australia
Author for correspondence: Jordan O Hampton, email: jordan.hampton@murdoch.edu.au

[OFFICIAL]

Table A. Median chase time, time to insensibility and total time (interquartile range) for aerial culling of fallow deer with four ammunition types and for all ammunition types combined.

Firearm/ammunition	Chase time	Time to insensibility	Total time
.308 rifle	1 min 17 s (1 min 5 s, 1 min 30 s)	9 s (8 s, 11 s)	1 min 27 s (1 min 13 s, 1 min 41 s)
00 Buck	1 min 12 s (1 min 0 s, 1 min 25 s)	12 s (10 s, 14 s)	1 min 24 s (1 min 10 s, 1 min 38 s)
1 Buck	1 min 18 s (1 min 5 s, 1 min 31 s)	12 s (9 s, 14 s)	1 min 30 s (1 min 15 s, 1 min 45 s)
4 Buck	1 min 29 s (1 min 15 s, 1 min 44 s)	10 s (9 s, 12 s)	1 min 40 s (1 min 23 s, 1 min 56 s)
All	1 min 19 s (1 min 12 s, 1 min 26 s)	11 s (10 s, 12 s)	1 min 30 s (1 min 22 s, 1 min 37 s)

[OFFICIAL]
